# Effect of COVID-19 protective measures on the epidemiology characteristics of rotavirus, adenovirus, and coinfections among pediatric patients with acute gastroenteritis in Hangzhou, China

**DOI:** 10.1128/spectrum.04007-23

**Published:** 2024-02-12

**Authors:** Jianming Zhou, Yanhong Sun

**Affiliations:** 1 Department of Clinical Laboratory, National Clinical Research Center for Child Health, National Children’s Regional Medical Center, The Children’s Hospital, Zhejiang University School of Medicine, Hangzhou, China; Xinxiang Medical University, Xinxiang, China

**Keywords:** rotavirus, adenovirus, acute gastroenteritis, COVID-19, children

## Abstract

Human rotavirus (RV) and adenovirus (AdV) have been recognized as common enteric viruses associated with viral acute gastroenteritis (AGE) in children aged<5 years. However, with the transmission of coronavirus disease 2019 (COVID-19) has been suppressed due to various aggressive and effective anti-epidemic measures, the prevalence of other viruses has also been affected. Therefore, this study aimed to investigate the impact of COVID-19 on the epidemiological characterization of RV, AdV, and coinfections among children with AGE in a hospital in Hangzhou from 2019 to 2023. The overall changes, seasonal distribution, and age distribution of enteroviruses were analyzed based on 5 years of records. All data were analyzed using SPSS 27.0. A total of 102,049 samples were analyzed from January 2019 to August 2023, and among them 15,911 (15.59%) were positive specimens, 11,646 (11.41%) were RV-positive, 4,057 (3.98%) were AdV-positive, and 208 (0.20%) were coinfection. The positive rate among males was 15.54%, while among females was 15.66% with a male-to-female ratio of 1.42:1. There was no significant difference in the positive rates of enterovirus infection between males and females. Significant associations were found between the month group and RV/AdV infection, with RV detection peaking in winter (74.18%) and early spring (29.22%), while AdV has a high prevalence in summer (16.03%) and spring (12.71%). The age group was also found to be significantly associated with RV/AdV infection, with RV being most prevalent in the 1–3-year-old age group (16.99%), while AdV was highest in the 3–5-year-old age group (8.10%).

**IMPORTANCE:**

This study highlights the epidemiological changes of rotavirus (RV), adenovirus (AdV), and coinfections in children with acute gastroenteritis (AGE) before, during, and after coronavirus disease 2019 (COVID-19) periods. There was a highly statistically significant difference in the positive rates of RV-positive, AdV-positive, and coinfection (*P* < 0.001), indicating that RV remains the main pathogen causing AGE. It emphasizes the importance of continuous surveillance of RV and AdV at both local and global levels. Regular surveillance of prevalent rotavirus strains will facilitate the development of new inactivated rotavirus vaccines and aid in disease prevention and control.

## INTRODUCTION

Acute gastroenteritis (AGE) is the most common infectious disease syndrome in children, with 50% of cases caused by viruses, 15% by bacteria, and 10% by parasites. Among the viral causes, the most common ones are rotavirus (RV), adenovirus (AdV), norovirus, astrovirus (AstV), coronavirus (CoV), and some picornaviruses (1, 2).

RV, which belongs to the family Reoviridae, is currently classified into 35G and 50P genotypes based on nucleotide sequences of the VP4 and VP7 genes (3). RV is a well-known cause of acute gastroenteritis in children under the age of 5 years worldwide, particularly group A rotavirus infection (4). RV infections can affect not only the gastrointestinal tract but also other parts of the body, leading to symptoms that range from mild watery diarrhea or subclinical illness to severe, frequent diarrhea accompanied by vomiting, fever, dehydration, electrolyte imbalance, and even death (5). In some cases, RV infection can also result in extra-intestinal complications, such as various neurological disorders, hepatitis and cholestasis, type 1 diabetes, respiratory illness, myocarditis, renal failure, and thrombocytopenia (6). Studies have shown that RV is responsible for approximately 527,000 deaths annually, accounting for 29% of all deaths due to diarrhea in children <5 years of age (7).

AdV is a member of the Adenoviridae family, belonging to the mastadenovirus genus. It can be categorized into seven species, namely A–G. AdV is known to cause a wide range of clinical symptoms, including AGE, acute respiratory infections, acute conjunctivitis, keratoconjunctivitis, pharyngoconjunctival fever, and urinary tract infections (8, 9). In pediatric patients with AGE, the predominant serotypes of AdV are AdV-41 and AdV-40, the only members of AdV-F; other AdV species, such as AdV-A (serotypes 12, 18, and 31), AdV-B (serotypes 3, 7, 11, and 14), AdV-C (serotypes 1, 2, and 5), and AdV-D (serotypes 28, 29, 30, 32, 37, and 43–46), have also been associated with diarrhea, although less frequently (10–16).

The overall prevalence of rotavirus and adenovirus was 29.8% and 6.3% of tested samples, respectively, with 160 studies from 18 countries from 1980 to 2019, mainly the Middle East and North Africa region (17). RV and AdV dominated from February to April in Denmark, and 22% of the samples were positive for rotavirus in March (18). The prevalence of AdV was recorded (7%), followed by HRV (2%) in Saudi Arabia (19). Viral infections are still the leading cause of AGE in hospitalized children, and the use of rotavirus vaccines did not reduce the number of hospitalizations per year (20).

Due to the coronavirus disease 2019 (COVID-19) pandemic, various non-pharmaceutical interventions (NPIs) have been implemented to reduce the transmission of the coronavirus and have also had a significant impact on the prevalence of common respiratory viruses and enteroviruses (21–23). However, limited studies have been conducted on the epidemiological characteristics of RV and AdV in children with AGE before, during, and after the COVID-19 pandemic. Therefore, this present study aims to retrospectively analyze the epidemiological characteristics of RV and AdV in patients with AGE over 5 years.

## RESULTS

### Changes in the positive rate

**Fig 1:**
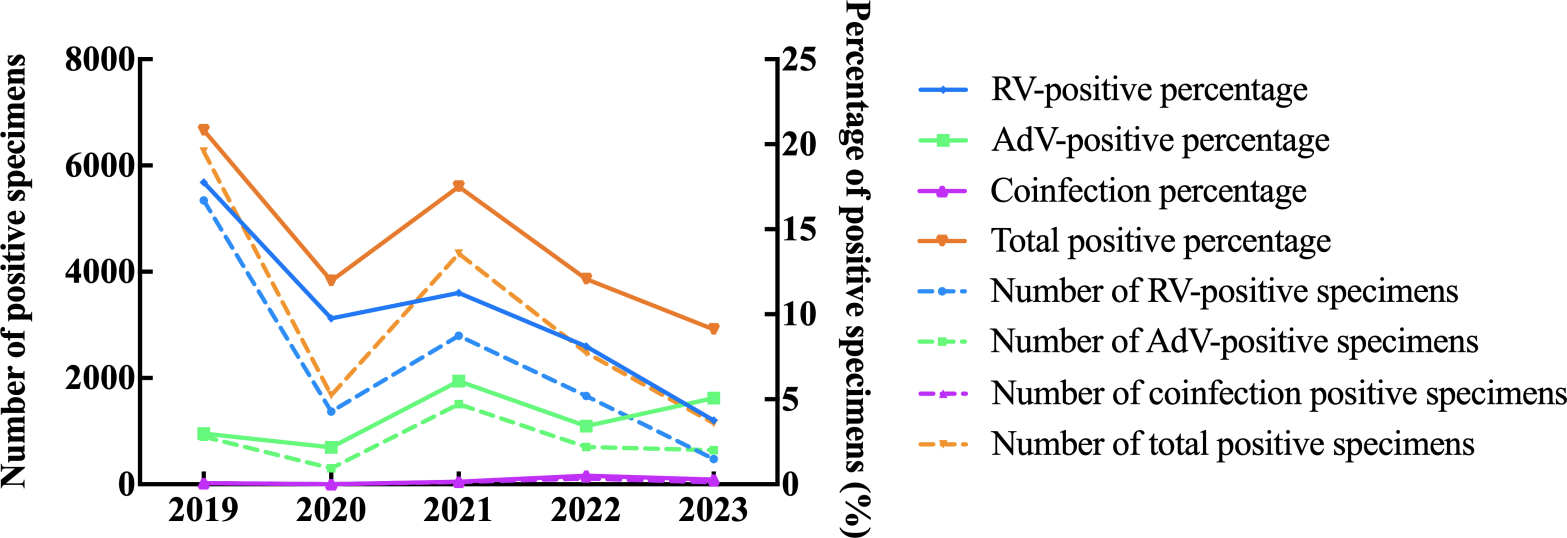
Year-on-year distribution of rotavirus, adenovirus, and coinfection positive specimen number and positive percentage.

The study analyzed a total of 102,049 stool specimens for the presence of RV and AdV. Out of these, 15,911 specimens (15.59%) tested positive for either RV or AdV (Table 1). A *χ*^2^ test was conducted to assess the significance of the difference in the number of positive specimens across the years 2019, 2020, 2021, 2022, and 2023. The total *χ*^2^ value was 1,432.33, with a *P* value less than 0.001, indicating that the difference in positive specimens between these years was highly significant. This suggests that the COVID-19 pandemic may had a significant impact on the number of positive tests over the 5 years. The overall positive rate, which represents the proportion of positive specimens out of the total tested, decreased from 20.83% before the COVID-19 pandemic to 11.96% in 2020, 17.50% in 2021, 12.07% in 2022, and 9.11% in 2023. This decline in positive rates indicates a reduction in the prevalence of RV and AdV infections during the COVID-19 epidemic (Fig. 1).

**TABLE 1:** Comparison of demographic characteristics and positive rate (%) of enteroviruses between the pre-COVID-19, COVID-19, and post-COVID-19 periods

	Pre-COVID-19	COVID-19 pandemic			Post-COVID-19			
Demographics	Jan 2019 to Dec 2019	Jan 2020 to Dec 2020	Jan 2021 to Dec 2021	Jan 2019 to Dec 2022	Jan 2023 to Aug 2023	Total	*χ*^2^ value	*P* value
Total positive specimens	6,262	1,678	4,345	2,477	1,149	15,911	1,432.33	<0.001
Total negative specimens	23,801	12,347	20,479	18,042	11,469	86,138		
Total tested specimens	30,063	14,025	24,824	20,519	12,618	102,049		
Positive rate	20.83%	11.96%	17.50%	12.07%	9.11%	15.59%		
Gender								
Male (total)	3,669/17,781	981/8,202	2,522/14,468	1,476/12,194	697/7,488	9,345/60,133	0.289	0.591
Female (total)	2,593/12,282	697/5,823	1,823/10,356	1,001/8,325	452/5,130	6,566/41,916		
Male (RV)	3,107/17,781	807/8,202	1,632/14,468	980/12,194	285/7,488	6,811/60,133	1.061	0.303
Female (RV)	2,234/12,282	562/5,823	1,164/10,356	686/8,325	189/5,130	4,835/41,916		
Male (AdV)	550/17,781	172/8,202	866/14,468	425/12,194	393/7,488	2,406/60,133	0.251	0.616
Female (AdV)	348/12,282	134/5,823	643/10,356	279/8,325	247/5,130	1,651/41,916		
Male (coinfection)	12/17,781	2/8,202	24/14,468	71/12,194	19/7,488	128/60,133	0.588	0.443
Female (coinfection)	11/12,282	1/5,823	16/10,356	36/8,325	16/5,130	80/41,916		
Virus detection, *n* (%)								
RV	5,341 (17.77)	1,369 (9.76)	2,796 (11.26)	1,666 (8.12)	474 (3.76)	11,646 (11.41)	11,845.0	<0.001
AdV	898 (2.99)	306 (2.18)	1,509 (6.08)	704 (3.43)	640 (5.07)	4,057 (3.98)		
Coinfection	23 (0.07)	3 (0.02)	40 (0.16)	107 (0.52)	35 (0.28)	208 (0.20)		

The gender distribution of the test cases showed that 15.54% of males (9,345 out of 60,133) and 15.66% of females (6,566 out of 41,916) tested positive for RV or AdV. The male-to-female ratio was approximately 1.42:1, indicating a slightly higher positive rate among males, although this difference was not statistically significant. Out of the total 11,591 positive cases, RV-positive accounted for 11,646, AdV-positive for 4,057, and coinfection for 208 cases. The positive rates of RV, AdV, and coinfection were found to be significantly different, indicating that RV remains the main pathogen responsible for AGE cases. Overall, the study suggests that the COVID-19 pandemic may had a significant impact on the prevalence of RV and AdV infections, leading to a decrease in positive rates. RV emerged as the primary causative agent of AGE cases during the study period. The findings also provide insights into the gender distribution of these infections, with a slightly higher positive rate observed among males.

### Changes in seasonality distribution

**Fig 2:**
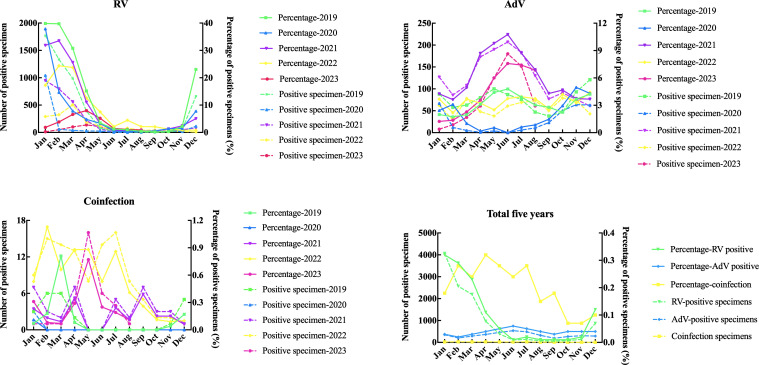
Seasonal distribution of rotavirus, adenovirus, and coinfection positive specimen number and positive percentage among pre‐COVID‐19, COVID‐19, and post‐COVID‐19 patients.

Tables 2 to 4 display the seasonality distribution of the positive number and percentage of RV, AdV coinfection, and total positive number and percentage according to the season. The corresponding data are represented graphically in Fig. 2. The months of December, January, and February are categorized as winter; March, April, and May as spring; June, July, and August as summer; and September, October, and November as autumn. A statistically significant relationship was observed between the month group and RV infection (*χ*^2^ value = 14,735, *P* < 0.001). The positive rate of RV was highest during the winter season, representing a total of 74.18% of cases (12.46% in December, 32.50% in January, and 29.22% in February). This was followed by early spring with a positive rate of 22.95% in March and 11.29% in April. During the COVID-19 pandemic, notably in 2020, there was a significant decrease in the number and percentage of RV-positive cases with a slight increase observed in 2021 and 2022. However, the trend continued to decrease in 2023 compared to the pre-COVID-19 period. Moving on to AdV, Table 3 presents the seasonality distribution of AdV. A highly significant relationship was found between the month group and AdV infection (*χ*^2^ value = 377.8, *P* < 0.001). The positive rate of AdV was highest during the summer season, accounting for a total of 16.03% of cases (6.29% in June, 5.49% in July, and 4.25% in August). Spring followed closely with a total positive rate of 12.71% (3.02% in March, 4.35% in April, and 5.34% in May). Similar to RV, the number and percentage of AdV-positive cases showed a decreasing trend in 2020 and an increase in 2021 and 2022 during the COVID-19 pandemic, as compared to the pre-COVID-19 period. This upward trend in AdV infections continued in 2023.

**TABLE 2:** Comparison of seasonal distribution of RV-positive between the pre-COVID-19, COVID-19, and post-COVID-19 periods

	Pre-COVID-19	COVID-19			Post-COVID-19	Total	*χ*^2^ value	*P* value
	2019	2020	2021	2022	2023		14,735	<0.001
January	1,770/4,442 (39.85)	1,043/2,754 (37.87)	949/2,979 (31.86)	291/1,684 (17.28)	12/649 (1.85)	4,065/12,508 (32.50)	601.85	<0.001
February	1,336/3,360 (39.76)	57/388 (14.69)	796/2,376 (33.50)	324/1,329 (24.38)	51/1,321 (3.86)	2,564/8,774 (29.22)	666.99	<0.001
March	988/3,210 (30.78)	39/497 (7.85)	561/2,194 (25.57)	505/2,126 (23.75)	101/1,533 (6.59)	2,194/9,560 (22.95)	416.75	<0.001
April	391/2,575 (15.18)	28/586 (4.78)	220/1,988 (11.07)	187/1,484 (12.60)	137/1,717 (7.98)	963/8,350 (11.29)	83.2	<0.001
May	79/2,274 (3.47)	25/746 (3.35)	61/1,941 (3.14)	114/1,519 (7.50)	108/2,082 (5.19)	387/8,562 (4.52)	50.17	<0.001
June	23/1,821 (1.26)	4/754 (0.53)	16/1,925 (0.83)	35/1,631 (2.15)	35/2,374 (1.47)	113/8,505 (1.33)	16.05	0.003
July	19/1,953 (0.97)	3/986 (0.30)	17/2,102 (0.81)	78/1,754 (4.45)	22/2,052 (1.07)	139/8,847 (1.57)	119.76	<0.001
August	7/1,553 (0.45)	5/1,299 (0.38)	10/1,883 (0.53)	42/1,957 (2.15)	8/890 (0.90)	72/7,582 (0.99)	41.83	<0.001
September	3/1,368 (0.22)	9/1,566 (0.57)	8/1,794 (0.45)	40/1,914 (2.09)		60/6,642 (0.90)	43.33	<0.001
October	7/2,100 (0.33)	23/1,694 (1.36)	25/1,945 (1.29)	23/1,879 (1.22)		78/7,618 (1.02)	13.8	0.003
November	56/2,534 (2.21)	19/1,291 (1.47)	49/2,036 (2.41)	19/2,271 (0.84)		143/8,132 (1.76)	19.73	<0.001
December	662/2,873 (23.04)	114/1,464 (7.79)	84/1,661 (5.06)	8/971 (0.82)		868/6,969 (12.46)	528.43	<0.001

**TABLE 3:** Comparison of seasonal distribution of AdV-positive between the pre-COVID-19, COVID-19, and post-COVID-19 periods

	Pre-COVID-19	COVID-19			Post-COVID-19	Total	*χ*^2^ value	*P* value
	2019	2020	2021	2022	2023		377.8	<0.001
January	88/4,442 (1.98)	67/2,754 (2.43)	127/2,979 (4.26)	62/1,684 (3.68)	8/649 (1.23)	352/12,508 (2.81)	46.18	<0.001
February	58/3,360 (1.73)	12/388 (3.09)	86/2,376 (3.62)	29/1,329 (2.18)	18/1,321 (1.36)	203/8,774 (2.31)	29.49	<0.001
March	63/3,210 (1.96)	5/497 (1.01)	108/2,194 (4.92)	78/2,126 (3.67)	35/1,533 (2.28)	289/9,560 (3.02)	52.1	<0.001
April	80/2,575 (3.11)	1/586 (0.17)	173/1,988 (8.70)	48/1,484 (3.23)	61/1,717 (3.55)	363/8,350 (4.35)	131.81	<0.001
May	100/2,274 (4.40)	4/746 (0.54)	190/1,941 (9.79)	38/1,519 (2.50)	125/2,082 (6.00)	457/8,562 (5.34)	140.14	<0.001
June	87/1,821 (4.78)	0/754 (0.00)	207/1,925 (10.75)	61/1,631 (3.74)	180/2,374 (7.58)	535/8,505 (6.29)	147.44	<0.001
July	76/1,953 (3.89)	6/986 (0.61)	183/2,102 (8.71)	69/1,754 (3.93)	152/2,052 (7.41)	486/8,847 (5.49)	119.46	<0.001
August	47/1,553 (3.03)	11/1,299 (0.85)	130/1,883 (6.90)	73/1,957 (3.73)	61/890 (6.85)	322/7,582 (4.25)	81.92	<0.001
September	38/1,368 (2.78)	23/1,566 (1.47)	77/1,794 (4.29)	50/1,914 (2.61)		188/6,642 (2.83)	24.84	<0.001
October	47/2,100 (2.24)	50/1,694 (2.95)	91/1,945 (4.68)	79/1,879 (4.20)		267/7,618 (3.50)	22.14	<0.001
November	93/2,534 (3.67)	64/1,291 (4.96)	76/2,036 (3.73)	74/2,271 (3.26)		307/8,132 (3.78)	6.72	0.08
December	121/2,873 (4.21)	63/1,464 (4.30)	61/1,661 (3.67)	43/971 (4.43)		288/6,969 (4.13)	1.26	0.74

**TABLE 4:** Comparison of seasonal distribution of coinfection between the pre-COVID-19, COVID-19, and post-COVID-19 periods

	Pre-COVID-19	COVID-19			Post-COVID-19	Total
	2019	2020	2021	2022	2023	
January	3/4,442	3/2,754	7/2,979	9/1,684	1/649	23/12,508 (0.18%)
February	6/3,360	0/388	3/2,376	15/1,329	1/1,321	25/8,774 (0.28%)
March	6/3,210	0/497	2/2,194	14/2,126	1/1,533	23/9,560 (0.24%)
April	2/2,575	0/586	7/1,988	13/1,484	5/1,717	27/8,350 (0.32%)
May	0/2,274	0/746	0/1,941	8/1,519	16/2,082	24/8,562 (0.28%)
June	0/1,821	0/754	0/1,925	14/1,631	6/2,374	20/8,505 (0.24%)
July	0/1,953	0/986	5/2,102	16/1,754	4/2,052	25/8,847 (0.28%)
August	0/1,553	0/1,299	2/1,883	8/1,957	1/890	11/7,582 (0.15%)
September	0/1,368	0/1,566	7/1,794	5/1,914		12/6,642 (0.18%)
October	0/2,100	0/1,694	3/1,945	2/1,879		5/7,618 (0.07%)
November	1/2,534	0/1,291	3/2,036	2/2,271		6/8,132 (0.07%)
December	5/2,873	0/1,464	1/1,661	1/971		7/6,969 (0.10%)

### Changes in the positive rate of age distribution

**Fig 3:**
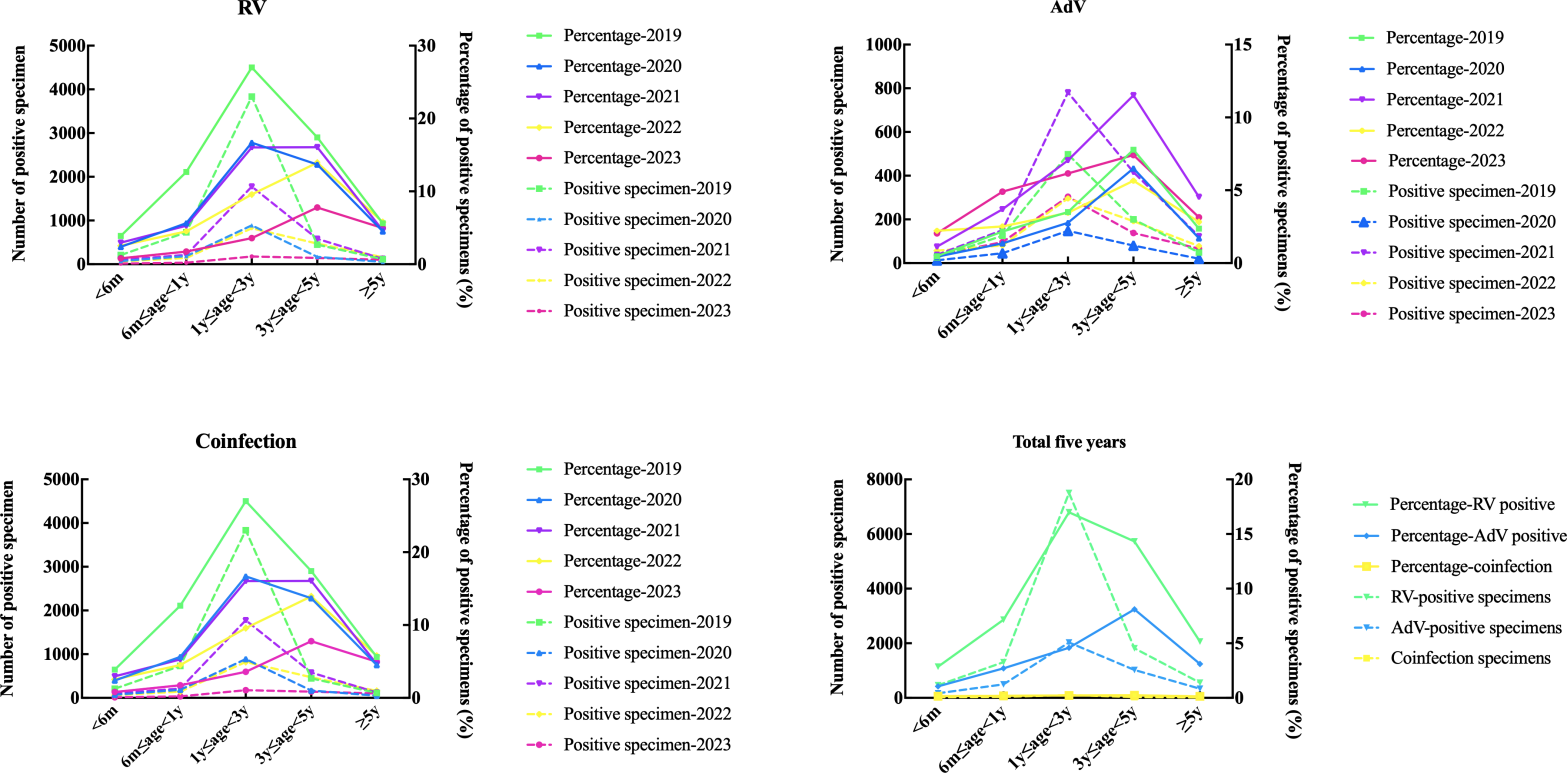
Age distribution of rotavirus, adenovirus, and coinfection positive specimen number and positive percentage among pre‐COVID‐19, COVID‐19, and post‐COVID‐19 patients.

The data presented in Fig. 3 and Tables 5 to 7 provide insights into the distribution of RV and AdV infections among different age groups. The statistical analysis conducted using the *χ*^2^ test revealed a highly significant association between age group and both RV and AdV infections (*P* < 0.001). For RV infection, the highest percent positivity was observed in the age group of 1–3 years (16.99%), followed by the age group of 3–5 years (14.32%). The median age of patients with RV infection was 2.1 years, ranging from 7 days to 16 years. Interestingly, the number and percentage of RV-positive cases showed a declining trend year by year from 2020 to 2023, even after the COVID-19 epidemic had almost ended. In the case of AdV infection, the highest percent positivity was observed in the age group of 3–5 years (8.10%), followed by the age group of 1–3 years (4.59%). The median age of patients with AdV infection was 2.3 years, ranging from 3 days to 17 years. Unlike RV infection, the number and percentage of AdV-positive cases showed a fluctuating trend from 2020 to 2023, with a decrease in 2020, followed by an increase in 2021 and 2022 compared to the pre-COVID-19 period. These findings emphasize the age-dependent susceptibility to both RV and AdV infections. The highest positivity rates were consistently observed in the younger age groups, indicating the need for targeted interventions, such as vaccination, for this population. It is noteworthy that even after the COVID-19 epidemic, the trends in RV and AdV infections appeared to be influenced by factors other than the pandemic. Please note that the above description is based on the given data and should be reviewed for accuracy and clarity.

**TABLE 5:** Comparison of the age distribution of RV-positive number and rate between the pre-COVID-19, COVID-19, and post-COVID-19 periods

	Pre-COVID-19	COVID-19			Post-COVID-19	Total	*χ*^2^ value	*P* value
	2019	2020	2021	2022	2023		3,377.8	<0.001
Median (min, max)	1.9y (5d, 13y)	1.9y (9d, 16y)	2.2y (4d, 13y)	2.5y (3d, 17y)	3.2y (7d, 11y)	2.1y (3d, 16y)		
<6m (*n*, %)	212/5,451 (3.89)	71/2,978 (2.38)	99/3,350 (2.96)	65/2,565 (2.53)	14/1,758 (0.80)	461/16,102 (2.86)	51.2	<0.001
6m ≤ age < 1y (*n*, %)	730/5,768 (12.66)	186/3,303 (5.63)	216/4,091 (5.28)	145/3,220 (4.50)	34/1,961 (1.73)	1311/18,343 (7.15)	417.21	<0.001
1y ≤ age < 3y (*n*, %)	3,835/14,205 (27.00)	891/5,342 (16.68)	1,776/11,072 (16.04)	824/8,585 (9.60)	177/4,945 (3.58)	7,503/44,149 (16.99)	1,978.9	<0.001
3y ≤ age < 5y (*n*, %)	4,48/2,572 (17.42)	168/1,228 (13.68)	580/3,610 (16.07)	473/3,396 (13.93)	145/1,861 (7.79)	1,814/12,667 (14.32)	94.58	<0.001
≥5y (*n*, %)	116/2,067 (5.61)	53/1,174 (4.51)	125/2,701 (4.63)	159/2,753 (5.78)	104/2,093 (4.97)	557/10,788 (5.16)	5.71	0.222

**TABLE 6:** Comparison of age distribution of ADV-positive between the pre-COVID-19, COVID-19, and post-COVID-19 periods

	Pre-COVID-19	COVID-19			Post-COVID-19	Total	*χ*^2^ value	*P* value
	2019	2020	2021	2022	2023		1,065.1	<0.001
Median (min, max)	2.2y (3d, 11y)	2.3y (6d, 8y)	2.4y (4d, 17y)	2.4y (3d, 14y)	2.2y (5d, 13y)	2.3y (3d, 17y)		
<6m (*n*, %)	26/5,451 (0.48)	13/2,978 (0.44)	38/3,350 (1.13)	57/2,565 (2.22)	36/1,758 (2.05)	170/16,102 (1.06)	78.58	<0.001
6m ≤ age < 1y (*n*, %)	125/5,768 (2.17)	45/3,303 (1.36)	151/4,091 (3.69)	81/3,220 (2.52)	96/1,961 (4.90)	498/18,343 (2.71)	79.97	<0.001
1y ≤ age < 3y (*n*, %)	498/14,205 (3.51)	148/5,342 (2.77)	781/11,072 (7.05)	296/8,585 (3.45)	304/4,945 (6.15)	2,027/44,149 (4.59)	284.88	<0.001
3y ≤ age < 5y (*n*, %)	200/2,572 (7.78)	80/1,228 (6.51)	416/3,610 (11.52)	192/3,396 (5.65)	138/1,861 (7.42)	1,026/12,667 (8.10)	89.83	<0.001
≥5y (*n*, %)	49/2,067 (2.37)	20/1,174 (1.70)	123/2,701 (4.55)	78/2,753 (2.83)	66/2,093 (3.15)	336/10,788 (3.11)	30.81	<0.001

**TABLE 7:** Comparison of age distribution of coinfection between the pre-COVID-19, COVID-19, and post-COVID-19 periods

	Pre-COVID-19	COVID-19			Post-COVID-19	Total
	2019	2020	2021	2022	2023	
<6m	3/5,451	0/2,978	5/3,350	15/2,565	2/1,758	25/16,102 (0.16%)
6m ≤ age < 1y	2/5,768	1/3,303	10/4,091	14/3,220	7/1,961	34/18,343 (0.19%)
1y ≤ age < 3y	14/14,205	2/5,342	22/11,072	49/8,585	16/4,945	103/44,149 (0.23%)
3y ≤ age < 5y	3/2,572	0/1,228	2/3,610	20/3,396	6/1,861	31/12,667 (0.24%)
≥5y	1/2,067	0/1,174	1/2,701	9/2,753	4/2,093	15/10,788 (0.14%)

### Changes in viral coinfections

The data presented show a trend in the number and rate of coinfections of RV and AdV over the years. In 2020, there was a decline in both the number and rate of coinfections compared to 2019, with three cases and a rate of 0.02% in 2020, as opposed to 23 cases and a rate of 0.07% in 2019. However, the trend reversed in subsequent years with an increase in both the number and rate of coinfections. In 2021, there were 40 cases with a rate of 0.16%, in 2022, there were 107 cases with a rate of 0.52%, and in 2023, there were 35 cases with a rate of 0.28%. Furthermore, when analyzing the seasonality distribution of coinfections, it was observed that the highest positive rates were recorded during spring with a total of 0.84%. Within the spring season, the rates were 0.28% in February, 0.24% in March, and 0.32% in April. The next highest positive rates were observed during summer with a total of 0.80%. In this season, the rates were 0.28% in May, 0.24% in June, and 0.28% in July. Additionally, the distribution of coinfections by age group was analyzed, revealing that the percent positivity of coinfections was highest in the age group of 3–5 years with a rate of 0.24%. The following highest rate was observed in the age group of 1–3 years with a rate of 0.23%. Overall, the data in our research indicate a fluctuating trend in the number and rate of RV and AdV coinfections, with variations across different years, seasons, and age groups. Whereas the detection data are single-center retrospective data, multi-center studies are planned in the future; hence, the results require further validation.

## DISCUSSION

In this study conducted in Hangzhou, China, the researchers investigated the impact of various interventions implemented to control the COVID-19 pandemic on the transmission of common enteroviruses, such as RV and AdV, in children with AGE. The interventions, which included social distancing, face masks, hand sanitation, travel restrictions, quarantine policies, and restricted hospital visits, were found to not only effectively reduce the spread of severe acute respiratory syndrome (SARS)-CoV-2 (the virus responsible for COVID-19) but also change the epidemic pattern of other enteroviruses (24). To evaluate the impact of the COVID-19 interventions, the researchers compared clinical data from January 2019 to August 2023. It was observed that the number and percentage of positive specimens for enteroviruses showed a significant decreasing trend during the COVID-19 pandemic, particularly in the year 2020. There is a slight rebound in RV and AdV cases in 2021 and 2022, which may be due to a short relaxation period in 2021 and 2022 after tense NPIs prevented and controlled virus transmission, and children had more opportunities for cross-infection in kindergarten or social activities. Similar results can be found in the Chinese literature (25). Even after the relaxation of COVID-19 restrictions in 2023, the prevalence of enteroviruses remained low, which could be attributed to an increased awareness of social distancing and hygiene practices among the population. The decline in both testing numbers and positive rates following the COVID-19 outbreak may not only be directly related to the measures taken to control COVID-19, but also to a decrease in the total number of outpatient visits, inpatient visits, and a continuing decline in the birth rate (26). In conclusion, the study findings suggest that the interventions implemented for COVID-19 have had a significant impact on reducing the prevalence of common enteroviruses, such as RV and AdV, to a certain extent. These results highlight the importance of continuing to maintain practices such as social distancing and hygiene measures even after the COVID-19 pandemic to minimize the transmission of various infectious diseases.

RV infection is primarily transmitted through the fecal-oral route, but there is also evidence suggesting the possibility of respiratory transmission (27). The prevalence of RV and AdV infections varies across seasons and age groups worldwide. In temperate climates, RV infections tend to peak in the colder winter months and show clear seasonality. In tropical climates, however, RV infections can occur throughout the year, and the seasonality is less pronounced. Interestingly, it has been observed that higher temperatures and higher relative humidity are associated with fewer hospitalizations for rotavirus infection (28). In Hangzhou, which is located in the subtropical monsoon area and has a subtropical monsoon climate, our study found that the positive rate of RV was highest in the winter and spring months. The rate began to rise in December, peaked in January, and continued into February and March before declining in April. On the other hand, AdV infection rates were highest in the summer. The rate began to rise in May, peaked in June, and continued into July and August. It is important to note that the seasonal characteristics of AdV incidence may differ in each study due to variations in regional climate, sample size, and study duration. Overall, our findings highlight the seasonal patterns of RV and AdV infections in Hangzhou, providing valuable insights into the epidemiology of these viruses in the region (29).

Our study revealed that RV infections were most common in children aged 1–3 years, followed by those aged 3–5 years, and then the group aged 6 months to 1year. The positivity rate of RV was lower in patients under 6 months and those over 5 years old. On the other hand, AdV infections were most common in children aged 3–5 years, followed by those aged 1–3 years, and then the group over 5 years old. The positivity rate of AdV was lower in patients under 6 months and those aged 6 months to 1year. These findings indicate that the highest prevalence of RV and AdV infections occurs in children under 5 years old. This could be because children in this age group may not have developed good personal hygiene habits. Children under 1-year-old often put unclean objects into their mouths, or they may directly put their hands and feet into their mouths. Children aged 1–3 years often touch various unsanitary surfaces with their hands, leading to the ingestion of the virus. Additionally, children aged 3–5 years may be more susceptible to enterovirus infections due to increased social interaction after attending nurseries or kindergartens. In conclusion, our study highlights the age group under 5 years as being at the highest risk for RV and AdV infections. These findings emphasize the importance of promoting proper hygiene practices in young children to reduce the transmission of these viruses. Similar studies have found that the transmission of common enteroviruses, especially RV and AstV, may be interrupted by COVID-19 NPIs in 2020 in Kenya. As governments canceled most COVID-19 NPIs after 2020, patterns of enterovirus transmission appear to return to pre-pandemic levels (30). RSV prevalence decreased substantially following the implementation of NPIs to mitigate the COVID-19 pandemic but later rebounded (31).

RV is known to be a leading cause of severe infantile diarrhea worldwide. It is responsible for a wide range of diseases with complex serotype specificities, including neurological disorders, hepatitis, cholestasis, type 1 diabetes, respiratory illness, myocarditis, renal failure, and thrombocytopenia. In recent years, there has been an increasing number of RV-related extra-intestinal clinical manifestations (6, 32–34). Vaccines have proven to be one of the most successful preventive measures in history, especially oral vaccines (35). Currently, two live attenuated rotavirus vaccines are available worldwide (36). However, the efficacy of these vaccines is relatively low, highlighting the need for the development of a highly effective rotavirus vaccine. Recent advancements in developing an inactivated rotavirus vaccine indicate a significant progress towards disease prevention and control (37). AdV, on the other hand, is usually carried by asymptomatic individuals, and the contagiousness of asymptomatic carriers is generally lower than that of symptomatic individuals (38). However, our analysis in this study suggests that further preventive measures should be taken to control the transmission of viruses with asymptomatic carriers, particularly AdV, which can affect young infants and children. Therefore, it is crucial to continue monitoring RV and AdV diligently in children. Regular monitoring of prevalent RV and AdV strains can help in better adjusting vaccines to meet the specific needs of patients.

There are several limitations to be noted in our study. Firstly, the data collected were limited to a single hospital, which may not represent the overall population. It would have been more convincing if data from multiple hospitals or sources were collected to increase the generalizability of the findings. Secondly, we only focused on RV and AdV and did not include other enteroviruses such as norovirus, astrovirus, coronavirus, and some picornaviruses. This limits the comprehensiveness of our study as these viruses can also be significant causes of gastrointestinal illness. Future research should consider including a broader range of enteroviruses. Thirdly, we did not perform genotyping of the RV and AdV samples. Genotyping can provide valuable information about the specific viral strains circulating in the population and aid in understanding disease transmission and evolution. Additionally, studying drug-resistance genes in these strains would be beneficial in informing treatment options. We recommend that future studies include RV genotyping and explore drug resistance genes to enhance our understanding of these viruses. Lastly, the major constraint of our study was the lack of financial resources and time. Limited funding and time may have restricted the scope and depth of the study, as well as the ability to gather data such as norovirus, sapovirus, and astrovirus from multiple sources or perform additional analyses. RV and AdV have been recognized as common enteric viruses associated with AGE in children based on the previous literature; and hence, RV and AdV were decided as the main subjects of study. It is important to allocate adequate resources to future research to overcome these limitations and provide more robust results.

### Conclusion

From 2019 to 2023, the epidemiological characteristics of rotavirus, adenovirus, and coinfections have shown some interesting trends. Before the COVID-19 pandemic, the overall positive rate for these viruses was 20.83%. However, in 2020, during the pandemic, this rate dropped significantly to 11.96%. In the following years, there was a slight increase in positive rates, with 17.50% in 2021, 12.07% in 2022, and 9.11% in 2023. The positive rates of enterovirus infection did not show any significant difference between males and females. This suggests that both genders are equally affected by these viruses. Among the three viruses, rotavirus remains the main pathogen causing AGE. The detection rate of RV was highest during winter and early spring. On the other hand, AdV had a high prevalence during summer and spring. When it comes to age groups, the percent positivity of RV was highest among children aged 1–3 years. This indicates that young children are more susceptible to RV infections. On the other hand, AdV infections were most common in children aged 3–5 years. It is worth noting that the measures adopted during the COVID-19 pandemic had a significant effect on the prevalence of common enteroviruses in China. Due to a series of quarantine measures and other preventive strategies, the number of enterovirus-positive cases and the positive rate both declined. This suggests that the implemented measures were successful in reducing the spread of these viruses. Overall, these epidemiological characteristics highlight the impact of the COVID-19 pandemic on the prevalence of rotavirus, adenovirus, and coinfections. The positive rates decreased during the pandemic but showed some fluctuations in the subsequent years. The timing of peak prevalence varied for RV and AdV, with RV being more common during winter and spring, and AdV during summer and spring. Understanding these trends can help public health officials and healthcare providers in developing targeted prevention and control strategies.

## MATERIALS AND METHODS

**Fig 4:**
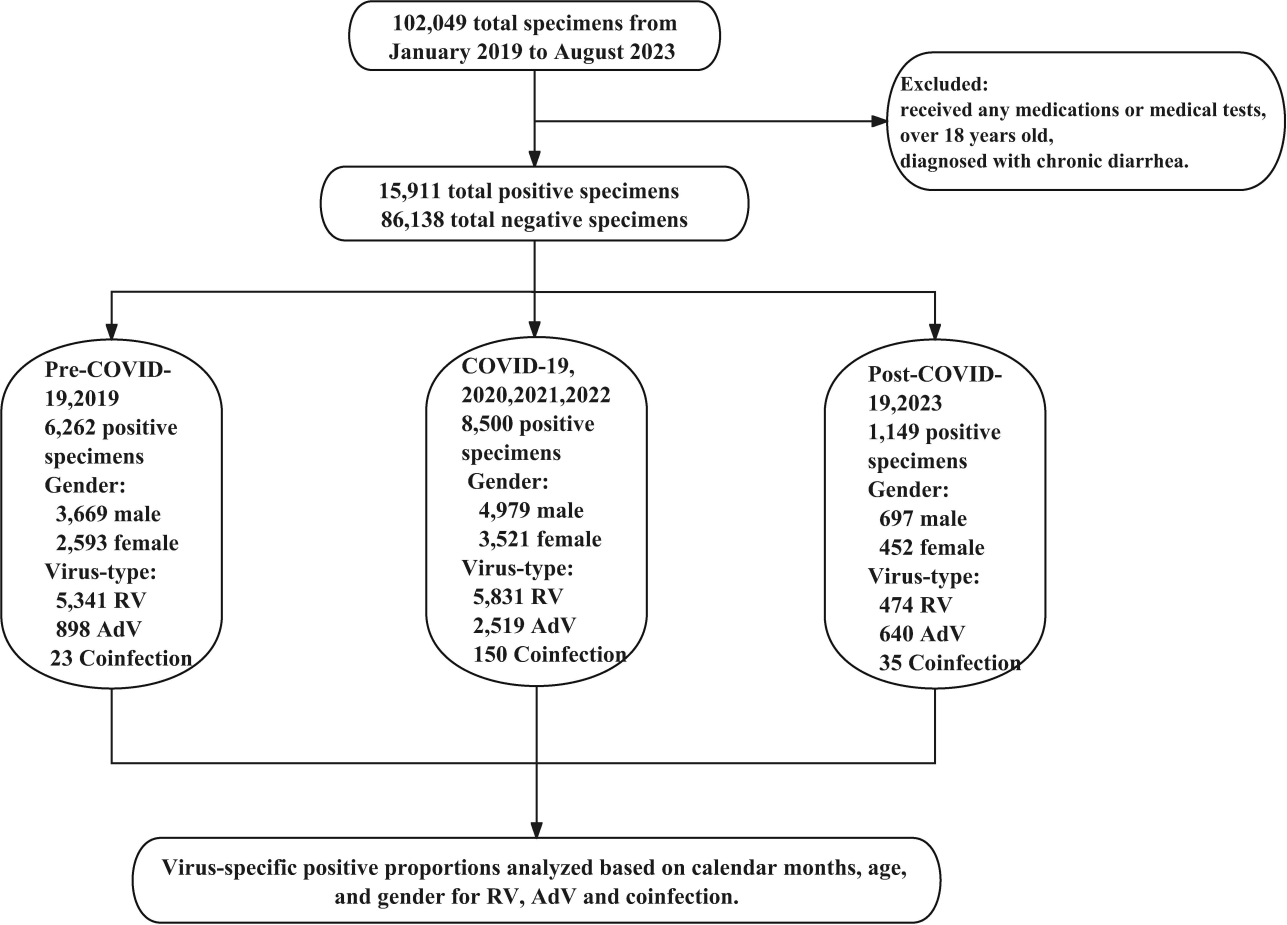
Flowchart of the methodology.

In this study, we collected laboratory and clinical data from both outpatients and inpatients aged less than 18 years who were diagnosed with AGE at the Children’s Hospital of Zhejiang University School of Medicine between January 2019 and August 2023 (Fig. 4).

Specifically, we analyzed the numbers of positive specimens for the RV, AdV, and coinfections. AGE was defined as the occurrence of three or more loose, watery, thin stools with a pasty texture or the presence of mucous stools per day, accompanied by symptoms such as vomiting, abdominal pain, fever, and lasting for less than 2 weeks (39). All subjects were classified into five age groups: under 6 months, 6 months to 1year, 1–3 years, 3–5 years, and older than 5 years. Additionally, we compared the monthly positive rates of RV and AdV. The demographic and clinical data of all enrolled patients were collected from their electronic medical records.

### Specimen collection and detection

Fecal specimens were collected from both outpatient and hospitalized children with diarrhea or fever by trained staff using clean containers. These samples were promptly sent to the clinical laboratory for enterovirus testing. The testing for RV and AdV was conducted using a chromatographic immunoassay method with an antigen test kit provided by Abon Biopharm Co., Ltd. In the previous research, using quantitative real-time polymerase chain reaction (RT-qPCR) as the gold standard, the sensitivity and specificity of the latex agglutination test for detecting rotavirus A were 81.03% and 97.44%, and the corresponding values for detecting human adenovirus were 76.27% and 94.19%, respectively (40). The well plate used for testing consisted of four main parts: a sample well plate, a control line, a rotavirus test line, and an adenovirus test line. To perform the test, approximately 50mg of fecal sample was mixed with 1mL of sample extraction reagent. Then, two drops (around 80 µL) of the sample mixture were added to the sample well plate. After an incubation period of 10–20 minutes, the test results were interpreted as follows:

- If both the control line and the rotavirus test line turned blue, the sample was considered positive for rotavirus.
- If the control line was blue and the adenovirus test line was red, the sample was determined as positive for adenovirus.
- If the control line was blue, the rotavirus test line was blue, and the adenovirus test line was red, the sample was considered positive for both rotavirus and adenovirus coinfection.
- If the control line did not change, regardless of the changes in the transmission virus detection line and adenovirus detection line, the determination on the well plate was deemed invalid.

Test results will be invalid after 20 minutes. RV or AdV lines in the test area may show different shades of color, but even very weak ribbon should be judged positive for the specified observation time, regardless of the color intensity of the ribbon. For the negative samples, if the clinician believes that the clinical symptoms are highly suspected of RV and AdV infection, RT-PCR is recommended for further diagnosis, other common pathogenic pathogens such as enterovirus, bacteria, fungi, parasites, etc. are also recommended to be tested for further diagnosis of the cause, and finally it is recommended to re-examine RV and AdV after 3–5 days.

### Statistical analysis

The study aimed to evaluate the virus-specific proportions among all children and adolescents who underwent fecal specimen testing, as well as among those with positive test results for RV and AdV. These proportions were analyzed based on calendar months, age, and gender for RV and AdV.

The study period was divided into three periods: the pre-COVID-19 period (1 January to 1 December 2019), the COVID-19 pandemic period (2 December 2019 to 31 December 2022), and the post-COVID-19 period (1 January 2023, onward). Descriptive statistical methods, including measures like median, frequency, and percentage, as well as statistical tests such as *χ*^2^ and Fisher’s exact tests, were used to compare the data between different groups. The Kruskal-Wallis *H* test, a nonparametric test, was used for multiple-group comparisons involving non-normal data. All statistical tests were two-sided, and a statistical significance level of *P* < 0.05 was considered significant. Highly significant results were defined as *P* < 0.001, while *P* > 0.05 indicated no significance. The IBM SPSS Statistics 27 software was used for data analysis.

## Data Availability

The original contributions presented in the study are all included in the article. Further inquiries can be directed to the corresponding author.
